# Heterogeneity of ventricular action potentials in neonatal rat cardiomyocytes and methodological aspects of patch clamp measurements

**DOI:** 10.3389/fphys.2025.1537345

**Published:** 2025-02-21

**Authors:** Pascal Syren, Anna Zlatopolskaia, Claus Bruehl, Axel Schöffel, Teresa Caspari, Chiara Heß, Norbert Frey, Dierk Thomas, Patrick Lugenbiel

**Affiliations:** ^1^ Department of Cardiology, University Hospital Heidelberg, Heidelberg, Germany; ^2^ HCR (Heidelberg Center for Heart Rhythm Disorders), University Hospital Heidelberg, Heidelberg, Germany; ^3^ DZHK (German Centre for Cardiovascular Research), Partner Sites Heidelberg/Mannheim, Heidelberg, Germany; ^4^ Department of Physiology and Pathophysiology, Heidelberg University, Heidelberg, Germany

**Keywords:** cardiac action potential, neonatal cardiomyocyte, patch clamp, methodological aspects cardiac action potential, cardiac action potential duration, induction current effect, membrane potential effect

## Abstract

Measurement of the ventricular action potential (AP) via whole-cell patch clamp is an important contributor to cardiac electrophysiological research. Neonatal rat ventricular cardiomyocytes (NRVCM) are a commonly used model, in particular for stressor- or drug-related questions. High variability of APs and individual methodological settings hinder comparison both in individual studies and, to an even greater degree, between different projects. This study aims to describe sources of AP heterogeneity in NRVCM related to patch clamp measurement with a focus on resolvable causes. Therefore, AP of NRVCM were induced in whole-cell configuration and measured in current-clamp mode. The effects of varying setup temperature, electrode resistance, resting- (RMP), respectively holding membrane potential (HMP), induction approach, current pulse duration and amplitude plus total assay duration were studied and compared to systematically analyzed literature. We analyzed the impact on different output parameters, namely, maximal upstroke velocity (dV/dt), maximal AP amplitude (APA) and AP duration at different percentages (XX%) of repolarization, APD_XX_. In a comparative literature research, we found that mean APD_90_ in between 27.0 and 560.7 ms (own data 59.7 ± 5.8 ms) were described, with high variability and likely non-Gaussian distribution. In this study, APD_90_, APD_50_ and APD_30_, are decreased at more negative RMP (respectively HMP) values. E.g., APD_90_ is shortened by ∼60% after lowering HMP from −70 mV to −90 mV) while dV/dt and APA are increased at a more negative HMP. Pulse duration in induction did not affect main AP parameters itself, but induction energy levels above 1.5-fold the threshold energy level increased APA, while APD_50_ and APD_90_ were shortened. During series of APs at 1 Hz, spike duration (APD_90_) decreased by ∼27%, with stable AP after the third repetitive AP. Spike duration did also decreased by ∼40% after prolongated measurements for 21 min, indicating degradation of electrophysiological properties. To improve data quality in NRVCM-APs, we suggest using a constant HMP, adjustment of current pulse amplitude to the individual cells’ threshold and the use of repetitive AP-inductions. Finally, we suggest the use of nonparametric statistical methods for statistical analysis. These aspects could reduce variability and lead to more reliable and comparable data.

## 1 Introduction

The patch clamp technique is the gold standard for functional assessment of single-cell electrophysiological properties since its introduction by Neher and Sakmann (Neher and Sakmann, 1976). It is utilized in various models, with neonatal rat ventricular cardiomyocytes (NRVCM) being frequently used for cardiac objectives due to their relatively robust nature, enabling prolonged culture duration, non-viral gene transfer, stressor- and drug-related assays (Louch et al., 2011; Chlopčíková et al., 2001). The ventricular action potential (AP), and its changes in various pathologies, are of great interest due to the high morbidity and mortality arising from ventricular arrhythmias, which constitute (in association with heart failure or myocardial infarction) a leading cause of death in the western population (Zeppenfeld et al., 2022). Therefore, the present work focuses on the heterogeneity of ventricular AP in NRVCM as measured by whole-cell patch clamp analysis, considering different methodological factors.

For patch clamp recording of APs in NRVCM, measurement of voltage over time data in whole-cell current clamp configuration is most often applied. Usually, ruptured patches (by application of negative pressure on the outside of the patch) are used. After resting membrane potential (RMP) measurement, some authors (Kilborn and Fedida, 1990; Lebeche et al., 2006; Hattori et al., 2012; Engels et al., 2015) report a current injection to reach a predefined holding membrane potential (HMP) of mostly −80 mV (−70 mV and −90 mV are also used). APs are typically induced by command current injections either with continuous repetition of single pulses at low frequency or in short trains (Kuryshev et al., 1999; Wickenden et al., 1997), often at higher frequencies.

Distinct parameters of the AP waveform are derived from the measured membrane potential. The maximal velocity of the depolarization (maximal upstroke velocity, dV/dt), characterizes the early phase of the AP, mostly defined by the density of *I*_Na_ (Attwell et al., 1979). The maximal AP amplitude (APA) is dependent on the relation of *I*_Na_ to the main early repolarizing currents *I*_Ca,L_, *I*_to,f_, *I*_to,s_ and, to smaller degree to *I*_Kr_, *I*_K2P_, *I*_f_ and *I*_NCX_ (Schmitt et al., 2014; Armoundas et al., 2003; Schram et al., 2002; Fenske et al., 2011). The subsequent early repolarization, plateau phase and late repolarization are most often described by the duration of the AP up to a defined percentage of repolarization. AP durations at 10%, 20%, 25%, 30%, 40%, 50%, 70%, 75%, 80%, and 90% of repolarization (APD_10_, APD_20_, APD_25_, APD_30_, APD_40_, APD_50_, APD_70_, APD_75_, APD_80_ and APD_90_) are described [e.g., (Zhang et al., 2019; Song et al., 2012; Korhonen et al., 2009; Fantini et al., 1990; Zasadny et al., 2020; Kang et al., 1995; Guo et al., 2007; Guo et al., 1996; Wickenden et al., 1997)], with APD_30_, APD_50_ and APD_90_ being slightly more widely used. APD of low percentages of repolarization (APD_10_ to APD_30_) are used to describe the early repolarization. APD_50_ is used to describe the plateau phase and the later APDs are used to describe the repolarization. Further parameters have been developed to describe and differentiate different shapes of APs. This includes solely visual characterization of the waveform as triangular or plateau-shape (Wu et al., 2013; Voitychuk et al., 2011; Bahrudin et al., 2011), but also quantification methods, e.g., the relation between maximal downstream velocity at plateau and repolarization phase (Ma et al., 2011; Syren, 2021a). There is, however, no common standard in reporting these AP properties. Most studies also report the RMP which is usually relatively stable and reflects mostly the density and reversal potential of potassium currents at rest (Bernstein, 1868; Bernstein, 1902).

NRVCMs have, beside their already mentioned advantages, several limitations when it comes to comparability with human (Varró et al., 2021) and adult rat physiologic properties. First, even slight differences in pregnancy duration and age of the neonatal rats at euthanization affect the differentiation process. APD decreases with increased differentiation of NRVCM, likely influenced by reduced *I*_K1_, *I*_to_ and increased *I*_f_ at an early differentiation stage (Kilborn and Fedida, 1990; Cerbai et al., 1999). Commonly, NRVCM utilized for patch clamp originate from animals in between 2.5 ± 0.37 days of postnatal age (Supplementary Table S1). Increased culture duration also shortens the AP and increases maximal upstroke velocity (dV/dt), likely by increased on channel expression and density, increased amplitude of *I*_to_ and decreased *I*_Ca_ in long-term culture (Guo et al., 1996; Meiry et al., 2001; Fast and Kléber, 1993; Kamiya et al., 1999; Wickenden et al., 1997). Culture durations for patch clamp analysis vary mostly in between 1 and 5 days, while APs from NRVCM after as long as 15 days of culture have been described (Guo et al., 1996). In culture, both spontaneously beating and quiescent NRVCM appear, without differences in APs (Zhang et al., 2010). If mentioned in literature, there is no clear preference in patch clamp assays for either cell type, with beating (Fantini et al., 1990; Gaughan et al., 1998; Michels et al., 2008; Wu et al., 2013; Sun et al., 2015; Zhao et al., 2016), quiescent (Snopko et al., 2007; Guo et al., 2007; Engels et al., 2015; Zhang et al., 2019; Oshiyama et al., 2022) or both (Guo et al., 1996; Rastan et al., 2005; McSpadden et al., 2012; Silva Dos Santos et al., 2023) cell types included in analysis. Culture medium and coating of surfaces affect NRVCMs (Viero et al., 2008; Parameswaran et al., 2013; Louch et al., 2011; Chlopčíková et al., 2001) after prolongated culture duration. In particular, high fetal bovine serum (FBS) concentrations are known to result in dedifferentiation (Viero et al., 2008). Furthermore, paracrine factors of cardiac fibroblasts (and likely the amount of fibroblasts in NRVCM culture) cause reduction of dV/dt and prolong AP due to reduced *I*_Na_, *I*_K1_ and *I*_to_ (Pedrotty et al., 2009).

In summary, biological variance, chosen methodological approaches, data analysis and choice of output parameters vary strongly and limit comparability in between available data. Furthermore, data on the impact of variation of the mentioned parameters are sparse. In the present study, whole-cell patch clamp measurements of NRVCM under various conditions were used to elucidate the respective influences on the resulting AP and contextualize these findings with existing literature.

## 2 Methods

### 2.1 Preparation and isolation of NRVCM

Animal experiments have been carried out in accordance with the Guide for the Care and Use of Laboratory Animals as adopted and promulgated by the U.S. National Institutes of Health (NIH publication No. 86–23, revised 1985) and with EU Directive 2010/63/EU, and the current version of the German Law on the Protection of Animals was followed. Experiments (institutional approval number T-24/22 and T-16/23) have been approved by the local animal welfare authority (Regierungspräsidium Karlsruhe, Karlsruhe, Germany).

Preparation and isolation of NRVCM was performed as described before (Syren, 2021b; Mages et al., 2024). Briefly, NRVCM were obtained from isolated hearts of 1–3 days old Wistar-Rats after euthanization by decapitation. Following thoracotomy, the hearts were excised, washed, and stored in ice cold ADS buffer (116.4 mM NaCl, 19.7 mM HEPES, 9.4 mM NaH_2_PO_4_, 5.6 mM D-glucose, 5.4 mM KCl, 0.8 mM MgSO_4_, pH 7.4 adjusted with NaOH) prior to enzymatic cell isolation. Vascular, non-cardiac (e.g., lung) and atrial tissue was removed. Tissue samples were mechanically dissected and enzymatically digested (0.6 mg/mL pancreatin (lot#SLBN 2032V, P7545), 0.5 mg/mL collagenase type 2 (lot#S5B15572, ACT 305 U/mg, LS004177); Sigma-Aldrich, Steinheim, Germany). The cell suspension was then filtered, and the number of fibroblasts was reduced through Percoll-gradient. Freshly isolated cells were then plated onto glass coverslips (P35G-0.170-14-C, MatTek, MA, United States) coated with collagen A (Sigma-Aldrich) and maintained in DMEM/F12 medium supplemented with 10% fetal bovine serum (GE Healthcare Technologies, Chicago, IL, United States) and 1% penicillin/streptomycin (Thermo Fisher Scientific, Waltham, MA, United States) at 37°C and 5% CO_2_ for at least 12 h at a density of 1.0 × 10^5^ viable cells per cm^2^. The medium was replaced after 24 h by long-term medium (containing 1% FBS).

### 2.2 Electrophysiological experiments

Induced APs of both quiescent and spontaneously beating single NRVCM were analyzed 12 h until maximal 4 days after isolation. Recordings were performed with an Axopatch 200B Amplifier (Molecular Devices, San Jose, CA, United States) and Signal software (version 4.11; Cambridge Electronic Design, Cambridge, United Kingdom) in ruptured patch whole cell configuration. Data were acquired at 20 kHz and filtered at 2 kHz using a four-pole Bessel low-pass filter. Pipettes were pulled from borosilicate glass capillaries (GB150-8P; Science Products, Hofheim am Taunus, Germany) using a DMZ Universal Puller (Zeitz Instruments, Martinsried, Germany) to achieve pipette resistances of 2.0–4.0 MΩ. Patch pipettes for cardiac AP recordings were filled with 130 mM KCl, 1 mM MgCl_2_, 5 mM EGTA, 5 mM MgATP, 10 mM HEPES, 10 mM NaCl (pH 7.2 adjusted with KOH). Extracellular solution consisted of 137 mM NaCl, 5.4 mM KCl, 1.8 mM CaCl_2_, 1 mM MgCl_2_, 10 mM D-glucose, 10 mM HEPES, and 2 mM sodium pyruvate (pH 7.4 adjusted with NaOH). Series resistance up to 30 MΩ was tolerated (with values mainly in between 4 and 10 MΩ). Cell capacitance was measured in voltage clamp utilizing 5 mV pulses at −80 mV HMP.

After formation of a seal resistance >1 GΩ and a stabilization period of 5 min after whole-cell-configuration, membrane potential was set to approximately −80 mV with a holding command current below 1,000 pA. Trains of 15 APs at 1 Hz stimulation rate were then elicited in current clamp mode by injection of brief current pulses (5 ms, at 1.0-fold the threshold up to 1.5-fold the threshold current strength, except specific experiments as stated in results). For repeated measurements, AP trains were elicited every 3 min. Recordings were carried out at 28°C ± 0.5°C or at 37°C ± 0.5°C respectively (custom build temperature controller). For more details on experimental procedures and data analysis see (Syren et al., 2021; Lugenbiel et al., 2021). Capacitance was derived by analogue compensation of the transient in response to square test pulses (amplitude 5 mV). Data analysis was performed using custom-written MATLAB routines (The MathWorks, Natick, MA, United States). For calculation of AP duration (APD) the absolute amplitude of the AP and the initial HMP/RMP was used as a reference point for determining the percentual repolarization. The timepoint of the maximal overshoot was set as start of APD-calculation to reduce effects of variability during AP induction.

### 2.3 Literature research and data extraction

A literature search was performed in PubMed to identify studies for a comparative review. The following search terms were used: “NRVCM” OR “NRCM” OR “neonatal rat cardiomyocyte” AND “AP” OR “action potential” and literature up to 17 August 2024 was reviewed. Secondary review was conducted by including selected references from initially selected studies as well as further review articles in the field. Articles presenting data exclusively from previously manipulated cells, non-single-cell assays, nonnumerical or graphical AP data were excluded from analysis.

Data from individual studies was directly included in analysis. In the case of no availability of numeric values, graphic values were included utilizing the software WebPlotDigitizer v.4.6 (https://automeris.io/WebPlotDigitizer, created September 2022 in Pacifica, California, United States by Ankit Rohatgi). Voltage over time data were derived from AP traces using the same software.

### 2.4 Statistics

Data are expressed as mean ± SEM. For better comparability with literature values, median and interquartile ranges are available on request. Statistical analyses were performed with GraphPad Prism 9.5.1 software (GraphPad Software, La Jolla, CA, United States). Statistical differences of unpaired, continuous variables were determined using Mann-Whitney *U* test, since normal distribution of APD cannot be assumed (compare Supplementary Material S3). Multiple comparisons of repeated measurements were performed using one-way ANOVA, as no interaction between independent nominal variables (i.e., animal groups) could be expected and the test is robust to non-normality (Blanca et al., 2023). If the hypothesis of equal means could be rejected at the 0.05-level, pair wise comparisons of groups were made using Dunnett’s multiple comparisons test, where appropriate. P < 0.05 was considered statistically significant. Due to confounding effects of biological variance, we did not perform statistical analysis on the literature data. For better comparability with literature data, data are presented as mean ± standard error of the mean (SEM). Median and interquartile ranges (IQR) for all data are available in the Supplementary Table S6.

## 3 Results

Firstly, an overview regarding AP heterogeneity in NRVCM is presented. Influences of technical aspects of patch-clamp methodology are presented in the following order: starting with setup temperature, electrode resistance, holding (respective resting) membrane potential, induction current frequency, duration and strength, and lastly duration of the experiment. In total, 464 publications were screened and data from 54 publications were included. For an in-depth overview of the chosen parameters and included data, compare Supplementary Table S1. Details of the original data analyzed in this work are provided in Supplementary Figure S1; Supplementary Table S2.

### 3.1 Action potential duration in NRVCM

**FIGURE 1:**
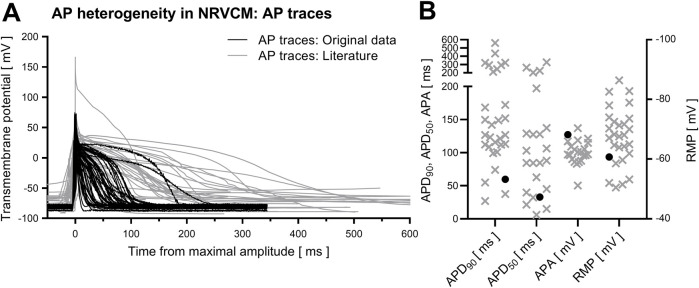
AP heterogeneity in NRVCM. Depicted are averaged traces of all analyzed APs of NRVCM [**(A)**, black] and APs in NRVCM from the analyzed literature [**(A)**, grey]. Mean values of APD_90_, APD_50_, APA and RMP from analyzed data [**(B)**, black circles] and literature [**(B)**, grey crosses] are shown.

AP in NRVCM are highly heterogeneous, both regarding AP-shape and APD. In our own experimental conditions (mostly consistent parameters regarding animal age at euthanization, animal handling, cell isolation, cell cultivation, duration of cultivation until electrophysiological analysis and settings in patch clamp analysis), APD_90_ ranged from 214.9 ms to 10.27 ms (mean 59.7 ± 5.8 ms, n = 48). In literature, mean reported APD_90_ measurements range from 27 ± 3.7 ms (Shi et al., 2020) up to 560.7 ± 117.3 ms (Hurtado et al., 2005). Similarly, APD_50_ varied strongly (own measurements: 32.8 ± 4.3 ms, published mean values from 6.1 ± 1.7 ms (Shi et al., 2020) up to 326.9 ± 65 ms (Hurtado et al., 2005)). Cell capacitance in our study was 21.2 ± 3.3 pF, while the reported (either digital or analogue) measured cell capacitance of NRVCM in literature ranges from 9.4 ± 2.4 pF (Kilborn and Fedida, 1990) up to 88.2 ± 6.1 pF (Kamiya et al., 1999), reflecting high variability in cell size. Exemplary reported APs of different studies and reported mean APD are depicted in Figure 1.

### 3.2 Influence of bath temperature

**FIGURE 2:**
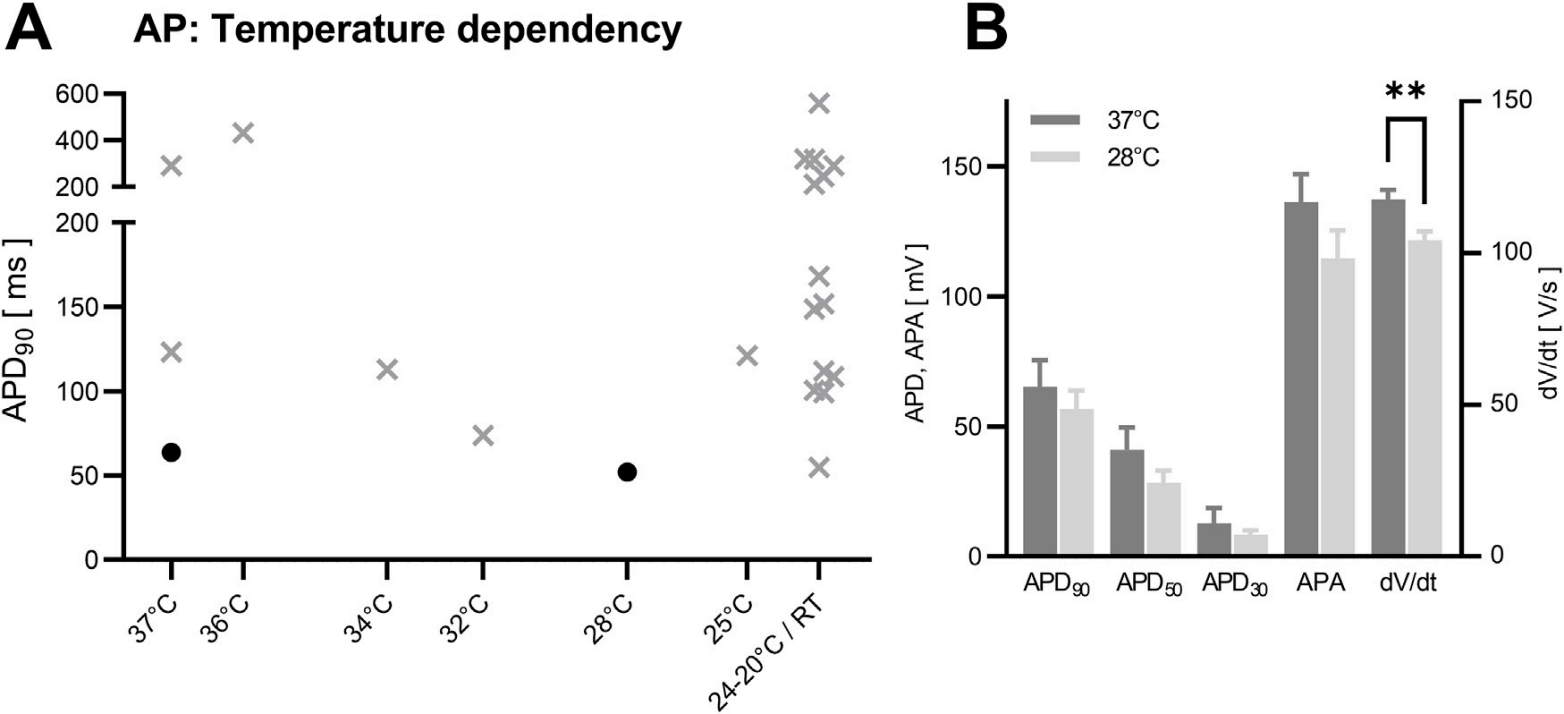
Temperature dependency in NRVCM-APs. Depicted are mean APD_90_ at different assay temperature [**(A)**, grey crosses: data from analyzed literature, black circles: original data]. Original data **(B)** showed no significant difference for APD_90_, APD_50_, APD_30_ and APA. A significant decrease was found for dV/dt in between 37°C and 28°C. data presented as mean + SEM, **: p < 0.01 (Mann-Whitney *U* test).

We did not observe significant changes of APD and APA between bath temperatures of 37°C (n = 17) and 28°C (n = 31) for APD_90_ (apparent difference of 13.0% ± 18.8% in between values at 28°C and 37°C, *p* = 0.468), APD_50_ (−31.0% ± 21.7%, *p* = 0.176), APD_30_ (−35.0% ± 38.1%, *p* = 0.915) and APA (−15.8% ± 12.2%, *p* = 0.189). dV/dt was significantly decreased by −11.5% ± 3.8% (*p* = 0.005) at the higher temperature of 37°C, as illustrated in Figure 2B. Qualitatively, no strong effect of temperature on APD was apparent in the literature (Figure 2A).

### 3.3 Influence of electrode resistance

We used a range of electrode resistance between 2,5 and 4 MΩ without qualitatively observable differences in AP waveform within this range. In the wide range from 0.4 to 1 MΩ (Liu et al., 2010; Yao et al., 1999) and between 10 and 15 MΩ (Korhonen et al., 2009) that is reported in literature, no effect on AP, including maximal upstroke velocity, is qualitatively apparent (Supplementary Figure S2).

### 3.4 Influence of holding/resting membrane potential

**FIGURE 3:**
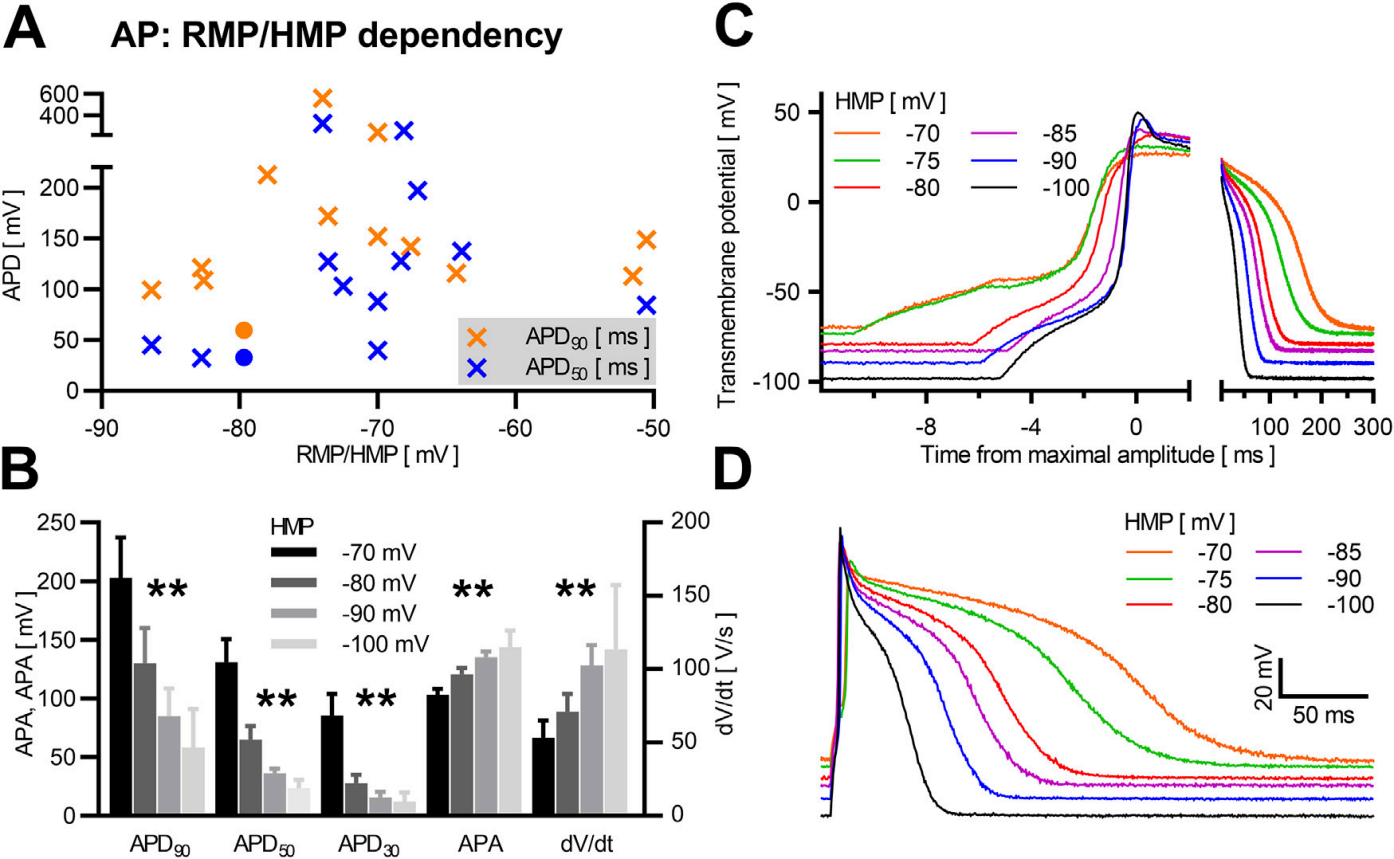
HMP/RMP dependency in NRVCM-APs Mean APD_90_ (orange) and APD_50_ (blue) with corresponding RMP/HMP are shown in **(A)** (crosses: literature data, circles: own data). Mean APD_90_, APD_50_, APD_30_, APA, and dV/dt with SEM of NRVCM (n = 7) are shown at different HMPs **(B)**. Exemplary AP-traces of the same neonatal rat ventricular cardiomyocyte at different HMP are shown with variable scale of the time-axis to illustrate early AP phases **(C)** and the whole AP **(D)**. *: p < 0.05, **: p < 0.01 (repeated measures ANOVA).

The reported resting membrane potential of NRVCM varied between −50.5 ± 3.1 mV (Snopko et al., 2007) and −86.4 ± 7.1 mV (Wickenden et al., 1997). In our assay, RMP varied between −27.0 mV and −81.4 mV (60.7 ± 2.9 mV), as illustrated in Figure 1B. The exact HMP during induction and measurement of the AP and the strength of current injections used for AP induction is rarely described. Apparently, −80 mV is the most widely used HMP (Metzele et al., 2011; Engels et al., 2015). Assuming that measurements were done at the reported RMP if not otherwise stated, we qualitatively observed a weak positive relation between RMP/HMP and APD_90_, as depicted in Figure 3A. In our measurements from single neonatal rat ventricular cardiomyocyte at different HMPs (n = 7), we observed a significant reduction of APD_90_, APD_50_ and APD_30_ at increasingly negative HMP. Furthermore, APA was increased significantly at more negative holding potentials, as was dV/dt (Figures 3B–D, data provided in Table 1).

**TABLE 1:** HMP/RMP dependency in NRVCM-APs.

HMP	APD_90_	APD_50_	APD_30_	APA	dV/dt
*p* value	0.001	<0.001	0.005	<0.001	0.011
−70 mV	202.6 ± 34.5 ms	130.9 ± 19.5 ms	85.4 ± 18.5 ms	103.0 ± 5.3 ms	53.2 ± 11.9 V/s
−80 mV	129.9 ± 29.9 ms	65.0 ± 11.3 ms	27.8 ± 7.1 ms	120.8 ± 5.5 ms	70.8 ± 12.5 V/s
−90 mV	84.8 ± 23.7 ms	36.2 ± 4.1 ms	15.5 ± 4.8 ms	135.3 ± 4.7 ms	102.5 ± 13.9 V/s
−100 mV	58.1 ± 13.4 ms	23.3 ± 2.8 ms	12.0 ± 3.2 ms	143.5 ± 5.9 ms	113.4 ± 18.0 V/s

Mean values ±SEM for APD_90_, APD_50_, APD_30_, APA and dV/dt at different HMPs (−70, −80, −90, and −100 mV) are shown. Respective *p*-values (One-way ANOVA with repeated measures) are shown.

### 3.5 Influence of induction current frequency, duration and amplitude

We varied induction pulse duration between 2 ms and 10 ms, which did not affect APD_90_, APD_50_, APD_30_, APA or dV/dt. Likewise, different pulse durations did not have major qualitative effects on AP shape. Only at high suprathreshold current amplitudes, prolonged pulses deformed the early phases of the AP due to overlap of the *I*_Na_-mediated depolarization with the induction pulse (Supplementary Figure S4, statistical data given in Supplementary Table S3).

**FIGURE 4:**
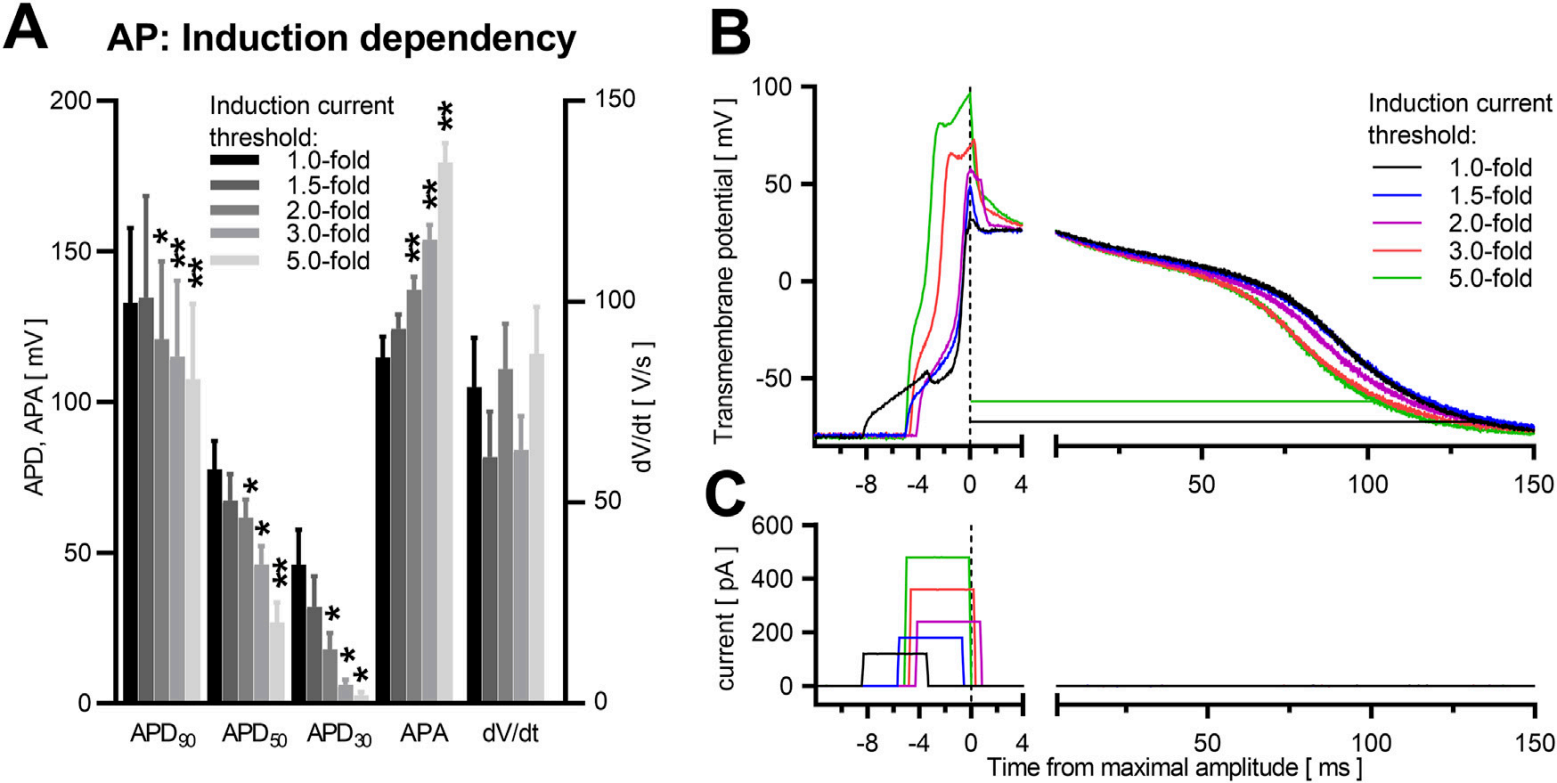
Induction energy dependency in NRVCM-AP. Mean APD_90_, APD_50_, APD_30_, APA, dV/dt with SEM close to the individual cells’ threshold (1.0-fold) and at 1.5-, 2.0-, 3.0- and 5.0-fold the threshold, are illustrated **(A)**. *: p < 0.05, n = 7. Exemplary AP traces are shown **(B)**. APD_90_ calculated at threshold energy level (black line) and at +400% (green line), are depicted. The timepoint at maximal AP amplitude for each trace is set to 0 ms. Corresponding rectangular induction current pulses are depicted in panel **(C)**. Statistical comparison in **(A)** with repeated measures ANOVA as main test, corrected for multiple comparisons with Dunnett’s test, +0% threshold as control column. n = 7. *: p < 0.05, **: p < 0.01.

In contrast, induction strength affects the early phase of the AP shape, in particular overshot, the maximal AP amplitude and, as a consequence, APA (Figures 4A,B). APA was increased by 56% ± 6.4%, *p* = 0.004 (Table 2) in identical NRVCM (n = 7) after increasing stimulation strength from AP induction threshold to 5.0-fold the threshold. Interestingly, no significant effect on dV/dt was apparent, although stimulation pulses still being active during the depolarization phase and at the timepoint of maximal upstroke velocity for 2.0-fold, 3.0-fold and 5.0-fold the threshold current strength (Figure 4C; Supplementary Figures S3A–C). Qualitive, different current strengths did not affect the plateau phase or late repolarization between threshold and around 2.0-fold the threshold current amplitude (Figure 4B; Supplementary Figures S3A–C), However, APD_90_, APD_50_, and APD_30_ were significantly shortened for 2.0-fold, 3.0-fold and 5.0-fold the threshold current strength compared to near-threshold stimulation (data shown in Table 2). Besides a true reduction of the late repolarization phase at high suprathreshold induction currents, these apparent effects were enlarged by the increase in APA by higher induction current amplitudes. The difference in calculated APD_90_ is illustrated in Figure 4B (black and green lines).

**TABLE 2:** Induction current dependency in NRVCM-APs.

Threshold	APD_90_	APD_50_	APD_30_	APA	dV/dt
*p* value (main test)	0.035	0.005	0.009	<0.001	0.437
1.0-fold	132.9 ± 24.9 ms	77.6 ± 9.5 ms	46.1 ± 11.5 ms	114.7 ± 6.9 ms	78.7 ± 12.3 V/s
1.5-fold	134.8 ± 33.6 ms	67.3 ± 8.7 ms	32.0 ± 10.1 ms	124.3 ± 4.7 ms	81.8 ± 15.0 V/s
*p* value	0.990	0.303	0.351	0.411	0.687
2.0-fold	120.9 ± 25.8 ms	61.5 ± 6.0 ms	17.9 ± 5.5 ms	137.2 ± 4.3 ms	83.2 ± 11.3 V/s
*p* value	0.026	0.035	0.032	0.007	0.527
3.0-fold	115.1 ± 25.2 ms	46.0 ± 6.1 ms	6.2 ± 1.7 ms	154.0 ± 4.8 ms	84.1 ± 11.1 V/s
*p* value	0.005	0.012	0.037	0.007	0.785
5.0-fold	107.5 ± 46.2 ms	26.8 ± 6.6 ms	2.6 ± 1.1 ms	179.5 ± 6.4 ms	87.0 ± 11.7 V/s
*p* value	0.003	0.004	0.033	0.004	0.621

Mean values ±SEM for APD_90_, APD_50_, APD_30_, APA and dV/dt at different induction current strengths (close to the individual cells’ threshold (1.0-fold) and at 1.5-fold, 2.0-fold, 3.0-fold and 5.0-fold the threshold) are shown. Respective *p*-values (One-way ANOVA with repeated measures as main test, corrected for multiple comparisons with Dunnett’s test) for comparison at 1.5-fold, 2.0-fold, 3.0-fold and 5.0-fold the threshold against 1.0-fold threshold are shown. n = 7.

### 3.6 Influence of repeated measurements and measurement duration

**FIGURE 5:**
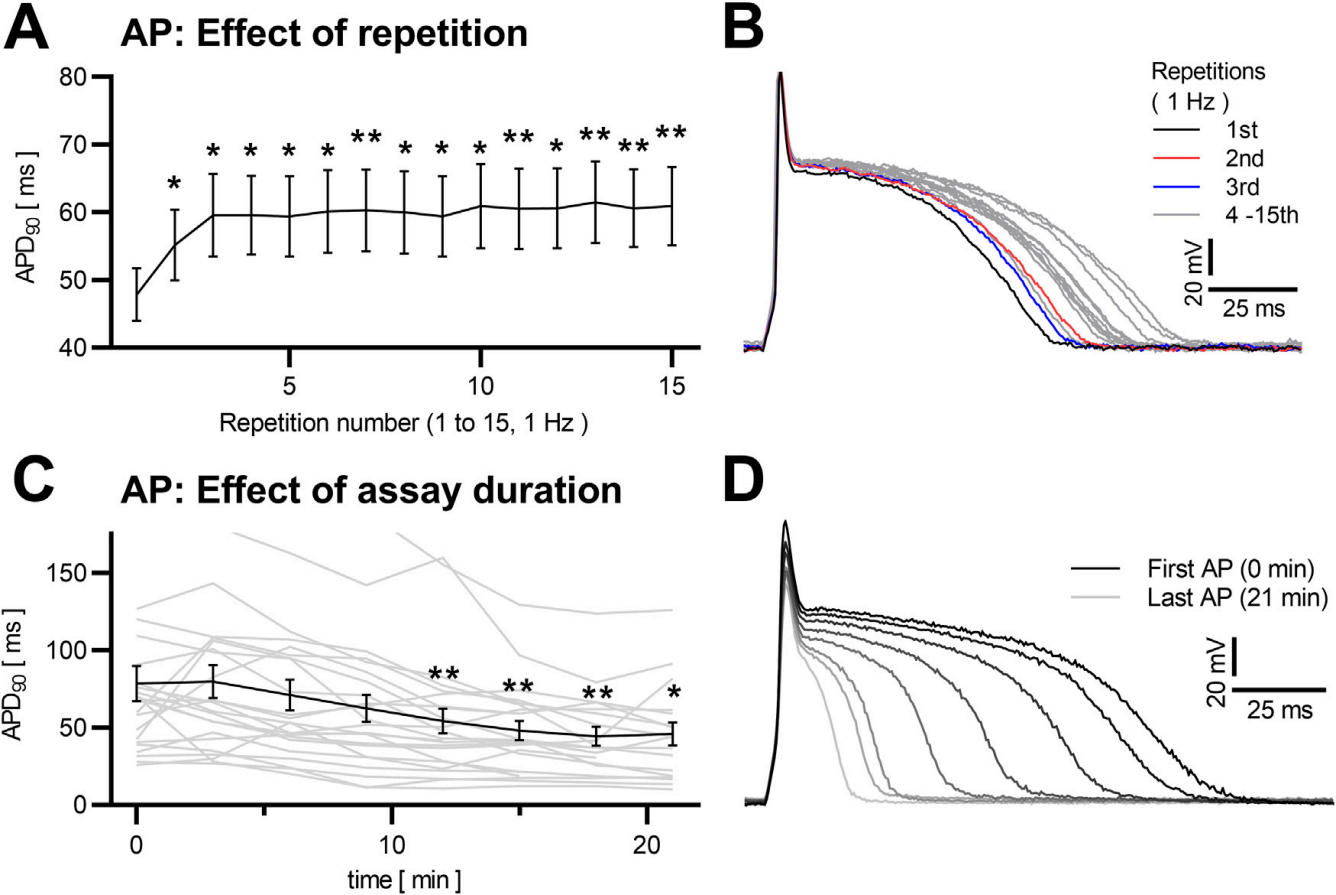
Dependency of NRVCM-AP to induction repetition and assay duration Mean APD_90_ of NRVCMs at 15 repetitive inductions (at 1 Hz stimulation frequency) are shown with SEM **(A)**. Exemplary traces (sweep 1 black, sweep 2 red, sweep 3 blue, sweep 4–15 grey) are depicted **(B)**. Mean APD_90_ (black) of NRVCM with AP-induction every 3 min over a timespan of 21 min (n = 23) and each cells individual data (grey) are shown **(C)**. Exemplary APs are shown (first AP black, increasingly brightening over the consecutive APs) in **(D)** *: p < 0.05, **: p < 0.01 (repeated measures ANOVA, corrected for multiple comparisons with Dunnett’s test, first AP as control).

Data from series of action potentials are often used instead of single AP analyses (Wickenden et al., 1997; Kuryshev et al., 1999), with sparse reported information on within-series differences in the analyzed literature. In our own recordings, we observed a AP prolongation mainly over the first three APs in consecutive trains of 15 APs at 1 Hz stimulation (*p* < 0.0001, multiple comparisons with +27.3 ± 6.3% for APD_90_, *p* = 0.007; +64.2 ± 15.4% for APD_50_, *p* = 0.010, n = 47, between the first and the 15th AP). After that initial prolongation, APs stabilized and did not show significant differences in APD_90_ (maximal difference of means from repetitions 3 to 15 5.9% ± 1.4%, *p* = 0.438) or APD_50_ (+7.1 ± 2.7%, *p* = 0.400), as illustrated in Figures 5A, B.

Concerning the overall duration of experiments, published data do not provide sufficient detail for a comparative analysis. In our own experience, measurements longer than 20 min begin with highly varying and generally prolongated APs during the initial ∼3 min (qualitative observation), followed by a relatively stable phase of ca. 10 min. Lastly, AP duration starts shortening (*p* < 0.0001, multiple comparisons significant with APD_90_–30.8 ± 8.2% at 12 min in comparison to the first measured APD_90_, *p* = 0.006, n = 23, and with APD_90_ −41.7 ± 12.0% at 21 min, *p* = 0.017, Figures 5C, D), until degeneration of the AP can be observed in cell death.

## 4 Discussion

High variability of the AP in NRVCM poses a relevant problem for data interpretation in individual studies themselves and to an even greater degree for the comparability between different publications. The data illustrated in Figure 1 with reported mean APD_90_ differing over more than one order of magnitude is combined with several reports of several clusters of different AP shape and consecutively different APD (Bahrudin et al., 2011; Wu et al., 2013). Possibly, these subgroups could arise from different cellular origin, e.g., epi- and endocardial localization (Voitychuk et al., 2011; Bahrudin et al., 2011), or could be caused by (de-)differentiation, as indicated by the influence of prolonged culture duration on different AP shapes (Wu et al., 2013). This data, indicating distinct subgroups, is consistent with several normality tests suggesting a non-Gaussian distribution of APD and APA in our data (Supplementary Section S2), further complicating statistical approaches and comparability. While the issue of significant variability in APD has been described earlier (Liang et al., 2015), and primarily biological causes like animal age, differentiation state of the NRVCM, culture duration (data from this work provided in Supplementary Section S6) and choice of culture media have already been discussed before (Viero et al., 2008; Parameswaran et al., 2013; Louch et al., 2011; Chlopčíková et al., 2001), the influence of patch clamp related parameters, to our knowledge, has not been discussed to the following extent before.

While a temperature of 37°C is usually applied for mammalian cell culture, except for specific purposes (Brown, 1990), the effect of temperature in the short time span of electrophysiological measurement is less clear. While measurement at 37°C reflects *in vivo* conditions best, it comes with the disadvantage of decreased cellular viability (and possible artificial APs of compromised cells), as described before (Chung and Campbell, 2013) and consistent with our (qualitative) observations. The significant increase of dV/dt at 37°C in our study is consistent with the finding of steep conduction increase of Na_V_1.5 (mediating *I*_Na_) at higher temperature (Milburn et al., 1995). We did neither observe significant changes of APA and APD in comparison of 28°C and 37°C, nor did observe a clear qualitative effect in literature data. This is in contrast to several works directly focusing on hypothermia, which are reporting a negative correlation of temperature with mainly QT in clinical observations and, less pronounced, of APD *in vitro*, attributing these changes mainly to decreased *I*_Ca_, *I*_K_ and *I*_K1_ (Kiyosue et al., 1993; Shah et al., 2006; Manzini et al., 1986; Dietrichs et al., 2019). Two possible reasons might explain these discrepancy: Firstly, temperature dependency of APD might be attributed to local cellular differences, as in particular an increase by over 300% in the dispersion of repolarization between different cardiac origins has been described in hypothermia (Piktel et al., 2011) and differences in hypothermia-associated APD changes between Purkinje fibres and ventricular myocardium have been described (Watanabe, 1975). Secondly, most studies addressing hypothermia-associated APD changes compare room temperature (20°C–23°C) or even lower temperatures with 37°C. Possible, the temperature dependency of the AP is not linear and more robust to changes closer to physiologic values. Supporting this hypothesis, a relative temperature insensitivity of calcium sparks above 25°C was described in cardiomyocytes (Fu et al., 2005) and guinea pig and ground squirrel cardiac APD_90_ showed a distinct change between 10°C and 24°C, which was not apparent in the guinea pig and reduced for ground squirrel from 24°C to 36°C (Herve et al., 1992). Under these observations, regular measurement at 37°C was recommended (Chung and Campbell, 2013). Our data support this approach, if the early phase of the AP (and in particular *I*_Na_) are within the scope of the research project. For analysis of cardiac repolarization, the decision is less clear and dependent on the assay design, where the faster cellular degradation and resulting artifacts under 37°C should be taken into consideration.

Lower electrode (and therefore access) resistance contributes to a lower series resistance, leading to artificial measurements in particular in voltage clamp (in particular for small currents (Armstrong and Gilly, 1992), while the effect size is debated and might be of low relevance for large currents (Gray and Santin, 2023)). For current clamp, RMP measurement is altered, likely due to increased and varying tip potentials, at last at vast differences in comparison to sharp microelectrodes (Li et al., 2004). Reported electrode resistance did not affect APA, APD or dV/dt significantly. Additionally, series resistance and its changes over the experiments’ duration can influence the RMP (Armstrong and Gilly, 1992) and possibly AP shape and duration. Input resistance reflects membrane resistance of the cell and might help in the categorization of NRVCM AP due to its sensibility to changes in potassium conductance (Cameron et al., 2000). Furthermore, its strong voltage dependency (Zaniboni et al., 2005) might influence cell capacitance and AP specifics observed in variable RMPs. The exact influence on AP heterogeneity in NRVCM of both series and input resistance are unknown and require further research.

Reported data on the RMP of NRVCM under experimental conditions also shows a wide variability with mean values between −50.5 (Snopko et al., 2007) and −86.4 mV (Wickenden et al., 1997). Whether current is injected during measurement to achieve a specific HMP or even to stabilize the initially measured RMP, is rarely mentioned, with −80 mV as most often described HMP (Metzele et al., 2011; Engels et al., 2015). In our experience, RMP under experimental conditions is seldomly stable and drops significantly over increased assay duration, making an increase of current injection necessary for stable HMP (data not shown). Furthermore, depolarized cells with initially low RMPs correlate with structurally compromised cells (Boyden et al., 1984) and should be discarded prior analysis (we suggest exclusion at RMP > −50 mV). In our study, we included such cells to better reflect the resulting data. The 5 cells included cells exhibited trendwise reduced APA of 97.6 ± 1.9 mV, APD_90_ of 46.6 ± 2.9 ms and APD_50_ of 25.5 ± 1.4 ms. APD is increased at a more positive RMP/HMP, while dV/dt and APA are negatively related. The observed behavior of dV/dt is likely highly influenced by the reduced steady-state channel availability of Na_V_1.5 at a more positive HMP, conducting *I*_Na_, whose peak current is elevated at a more negative HMP (Ruben et al., 1992; Walton and Fozzard, 1979). These kinetics of Na_V_1.5 might also explain the observed decrease of APA with a more positive HMP, with a reduced peak current of *I*_Na_ leading to a smaller overshoot and reduced dV/dt (Kodama et al., 1981; Coraboeuf et al., 1979). Prolonged APD in more positive HMP results most likely from altered kinetics of a multitude of ion channels active in repolarization. Block of delayed rectifier currents (*I*_K_) and the transient outward current (*I*_to_) with 4-AP respectively TEA reduced HMP-dependent AP prolongation in neurons (Gong et al., 2008), implicating HMP-dependent kinetics of the respective ion channel K_V_ subunits. Considering these observations, we suggest the use of a stable membrane potential either by stabilizing the measured RMP in single NRVCM for comparisons of APs in the same cell over time or, if comparisons in between different cells are of interest, a predefined HMP for all cells.

Mostly, current pulses are used for AP induction, with only rare occurrences of intrinsic APs being analyzed (33 out of 44 or 75% sources providing information on this topic in contrast to 7 (16%) analyzing only spontaneous APs and 4 (9%) analyzing both entities of APs). We found no significant differences between induced APs from spontaneously beating and quiescent NRVCM (compare Supplementary Figure S5; Supplementary Table S5), as was described earlier (Zhang et al., 2010). The choice of induction protocol is rarely reported. A rectangular pulse is most often utilized, but varies highly in regard to frequency (from 0.2 Hz (Voitychuk et al., 2011) up to 10 Hz (Hattori et al., 2012)), induction pulse duration (from 1 ms (Rastan et al., 2005) up to 10 ms (McSpadden et al., 2012)) and induction current strength. Since the latter is relative, different approaches are used, from application of a relative percentage above the individual cells threshold (varying from 1.1-fold the threshold (McSpadden et al., 2012) to 2.0-fold the threshold (Rastan et al., 2005; Sun et al., 2015)) over fixed current strength steps (Metzele et al., 2011; Oshiyama et al., 2022; Liang et al., 2015; Kang et al., 1995) to (high suprathreshold) fixed current strength (Voitychuk et al., 2011; Yang et al., 2021). While duration of the induction pulse (inside the chosen range from 2 up to 10 ms) did not influence the AP in our study, current durations above 10ms change kinetics of ion channels similar to altered HMP (e.g., described for *I*_Na_ (Walton and Fozzard, 1979)), and should therefore be avoided. In contrast, the total amplitude of the induction pulse directly influences the maximal upstroke and consecutively APA for currents above 1.5-fold the threshold energy *via* overlap of induction pulse and early AP. Interestingly, we did not observe a significant difference in dV/dt, even at induction pulses which are active over the whole upstroke phase of the AP. Probably, the conduction of Na_V_1.5 at the timepoint of dV/dt exceeds other currents to such an amount that their reaction to the induction pulse is irrelevant, as others have already pointed out the validity of dV/dt even under induced APs (Roberge and Drouhard, 1987). While the plateau phase and repolarization (including AP duration) seem to be relatively independent from induction until around 2.0-fold the threshold induction pulses, higher induction energies shorten the APD. First, the calculation method from APD, which is usually calculated from APA, contributes to these measurements, and artificially shortens APs at higher induction energy (and higher APA). Rarely described alternative APD calculations, such as fixed AP measurements at 0 mV and −60 mV (Liu et al., 2010), pose other problems, as described later. However, the APD prolongation at induction >2.0-fold the threshold cannot solely be explained by this confounding aspect. For several ion currents (in varying models, for following sources often own interpretations of I-V-relationships mentioned, since statistical tests for this question could not be found), voltage dependency with nonconstant channel kinetics beyond the regular maximal overshoot (at around +20 to +50 mV) has been described. For *I*_Kr_, the tail current is qualitatively higher at +60 and +80 mV compared to +0 and +20 mV (Haworth et al., 2014; Wu et al., 2015; Lu et al., 2014; Zhou et al., 1998). Similar results have been described for *I*_to_ (Lu et al., 2014) and *I*_Ks_ (Kaboua et al., 2022; Wu et al., 2015), including the tail current (Gou et al., 2018). while *I*_Ca,L,_ including its later components, is qualitatively steeply declining at values higher than +30 mV (Lu et al., 2014). Taken together, increased tail currents of positive currents contributing to repolarization (*I*_to_, *I*_Ks_ and *I*_Kr_) and decreased negative currents stabilizing the AP plateau (*I*_Ca,L_) at a very high overshoot induced by far suprathreshold induction pulses, shorten the APD. We suggest firstly the measurement of the induction energy threshold and following a slightly higher (e.g., 1.2-fold–1.5-fold the threshold) induction pulse energy. For projects addressing specifics of the early AP, e.g., *I*_Na_, we suggest an even stricter choice of induction pulse energy very close to the threshold, with a clear separation of induction pulse from the depolarization phase. Furthermore, we suggest omission of any distorted AP waveforms from analysis, since they are likely to originate from compromised cells or methodological issues.

After an early phase of unstable APs, for which an stabilization period of 5 min before measurement was proposed (Kang et al., 1995; Rastan et al., 2005), different approaches for pacing are applied. Steady pacing with frequencies between 0.2 Hz (Voitychuk et al., 2011) and 10 Hz (Hattori et al., 2012) were described. We observed faster cell degeneration and death during continuous pacing (qualitative observation) over prolonged time. This might be the reason for the application of repeated series of induced APs (Wickenden et al., 1997; Kuryshev et al., 1999). Single APs differ from continuous paced cells in the latter case, APs are frequency-dependent (Wickenden et al., 1997). In our data, APD stabilized after 3 repeated inductions at 1 Hz and stayed stable without significant changes for up to at least 15 repetitions. This effect, also termed “post-rest adaptation,” is well known in adult cardiomyocytes of several mammalian species (Becher and Ravens, 1982; Ishida et al., 1998; Bassani et al., 2004), but is less pronounced described in the adult rat (Bassani et al., 2004). Lastly, prolonged AP measurements are of interest, in particular concerning pharmacological effects on the AP. Considering the issue of high AP variability between different cells, this approach is likely superior in comparison to measurement of different drug-exposed NRVCMs. Unfortunately, prolongated measurements shorten APD significantly. Furthermore, prolongated measurements are more time consuming due to a high rate of cell death during measurements. In this study, 23 out of 48 NRVCM survived 20 min of measurement, and (whereas altered by drug exposure as additional stressor after 20 min, data not shown) only 6 out of initial 48 cells survived 50 min of measurement. We suggest an initial stabilization phase and afterwards either series of induced APs (in particular if a prolongated measurement period is planned) or steady pacing at a suitable frequency for measurement.

Data analysis of the voltage over time AP data is performed differently, with limitations to each approach. Due to variation between individual cells’ APs and the possibility of technical artifacts, mean or median values from several data traces in an AP series are often analyzed (Christ et al., 2001). While slightly different algorithms for smoothing, calculation data mean/median or maximal values are applied for the calculation of RMP, APA and dV/dt (Puddu et al., 2010; Árpádffy-Lovas and Nagy, 2023), we did neither find reported major issues with these values in literature nor did we observe such in our analysis. Calculation of APD, however, has several limitations. Most authors calculate APD from APA. Issues include firstly the heterogenous AP shapes in NRVCM (as discussed in the first paragraph), which influence in particular “early” APDs such as APD_10_ - APD_40_ due to the high potential differences in between maximal amplitude and plateau phase. Secondly, induction pulse energy influences APA and consecutively APD, as mentioned before. Furthermore, the starting point of measurement is sometimes calculated from maximal upstroke, e.g., in (Sallé et al., 2008) and our study, and alternatively from the begin of phase 0, e.g., (Avula et al., 2021; Burton and Cobbe, 2001). While the latter approach is including phase 0 and therefore the whole AP, it is vulnerable to artifacts arising from induction, with in particular “late” APD (APD_80_, APD_90_) being prone to inclusion of induction pulse variants in calculation (as observable in Figures 3C, 4B; Supplementary Figures S3A–C). As a further method, measurement of the AP width at fixed potential levels (e.g., 0 mV and −60 mV, roughly comparable to APD_30_ and APD_80_ (Liu et al., 2010)) is sometimes utilized. This approach is independent from induction pulse energy, but more influenced by different AP shapes and degeneration processes than the former variants. We recommend measurement from maximal upstroke until XX% of repolarization due to its robustness regarding AP shape, with compulsory awareness of the effect of induction with this calculation method and careful choice of suitable induction pulse energy. Concerning statistical approach and taking into account our findings of likely non-Gaussian APD- and APA-distribution (as discussed earlier), we suggest nonparametric tests, as already applied previously for NRVCM-APD (Oshiyama et al., 2022; Silva Dos Santos et al., 2023).

In summary, native cardiac APs of NRVCMs are highly variable in both shape and duration, hindering analysis and comparability. We suggest the use of a constant HMP, variable and closely adjusted to the individual cells’ threshold induction pulse energies and the use of repetitive AP-inductions. After an initial stabilization phase and repetitive induction series, a prolongated measurement period might be advisable, dependent on the objective. Setup temperature has to be considered if phase 0 and *I*_Na_ are regarded. For analysis, we suggest the measurement of APD from maximal upstroke and nonparametric statistic test for statistical analysis. Improved measurement consistency and consecutively reduced data variability might lead to more reliable and comparable results, improving electrophysiological cardiac research options.

## Data Availability

The raw data supporting the conclusions of this article will be made available by the authors, without undue reservation.
