# Supplementary material for: Heterogeneity of ventricular action potentials in neonatal rat cardiomyocytes and methodological aspects of patch clamp measurements
Source: Front Physiol. 2025 Feb 21;16:1537345. doi: 10.3389/fphys.2025.1537345 (PMC11885515; doi:10.3389/fphys.2025.1537345)
Supplement: Supplementary file 1 [file DataSheet2.pdf]

Heterogeneity of ventricular action potentials in neonatal rat cardiomyocytes and corresponding aspects of patch clamp measurements

Supplementary Material

1 Overview of methodological/measurement settings and resulting AP parameters in analyzed literature

Supplementary table 1: Overview of methodological/measurement settings and resulting AP parameters in analyzed literature
Selected parameters of methodological settings, experimental conditions and resulting AP parameters are summarized in this table. The number of publications reporting each parameter is given. In cases of separately described groups of NRVCM (e.g. isolated at 5 days of age vs. isolated at 1 day of age) in a single publication, each group was analyzed individually. In total, 61 NRVCM groups out of 54 individual publications were included. In the case of reported ranges in source literature (e.g. animal age at isolation in between 2 and 4 days), the mean value (3 in the given example) was used for each individual source. Values given with "greater/less than" were excluded from analysis.

	**Mean ± SEM (or stated parameter)**	**Number and percentage of reporting sources**	**Additional comments**
Animal age at isolation [d]	2.5 ± 0.4	59; 97%	1 source describing < 1 d
NRVCM time in culture [d]	3.8 ± 0.4	41; 67%	1 source describing > 2 d
Spontaneous beating [%]	42% yes, 37% no, 21% both	19; 31%	
NRVCM number	15.2 ± 2.4	51; 84%	Dots from scatter plots not counted
Electrode resistance [MΩ]	4.0 ± 0.3	43; 70%	1 source describing < 3 MΩ
Temperature [°C]	Compare main manuscript, section 3.2. and figure 2. Reported in 50 (82%) of the sources		
Analysis of:	Only induced APs (33/44, 75%), Only spontaneous APs (7/44, 16%), Both AP entities (4/44, 9%)		
Induction current [pA]	Mostly relative values or stated as "suprathreshold". Reported in 21 (34%) of the sources		
Induction duration [ms]	3.6 ± 0.6	27; 44%	2 sources applying a step protocol
Induction frequency [Hz]	1.3 ± 0.4	22; 36%	Use of sequences/trains vs continuous pacing is rarely reported
Capacity [pF]	50.9 ± 10.2	22; 36%	
RMP [mV]	-67.7 ± 1.9	27; 44%	In several cases potentially HMP (low variability, values close to -70 or -80 mV), reported as RMP
APD_{10} [ms]	12	1; 2%	
APD_{20} [ms]	16.2 ± 9.6	3; 5%	
APD_{25} [ms]	89.4 ± 11.2	4; 7%	

APD_{30} [ms]	88.7 ± 14.1	5; 8%	
APD_{40} [ms]	65.9	1; 2%	
APD_{50} [ms]	114.1 ± 18.0	22; 36%	
APD_{70} [ms]	79.8	1; 2%	
APD_{75} [ms]	215.0 ± 37.9	3; 5%	
APD_{80} [ms]	190.4 ± 18.3	10; 16%	
APD_{90} [ms]	174.6 ± 22.2	29; 48%	
APA [ms]	99.8 ± 3.9	21;34%	
dV/dt [V/s]	79.2 ± 13.0	12; 20%	

2 Overview of methodological/measurement settings and resulting AP parameters in the original data

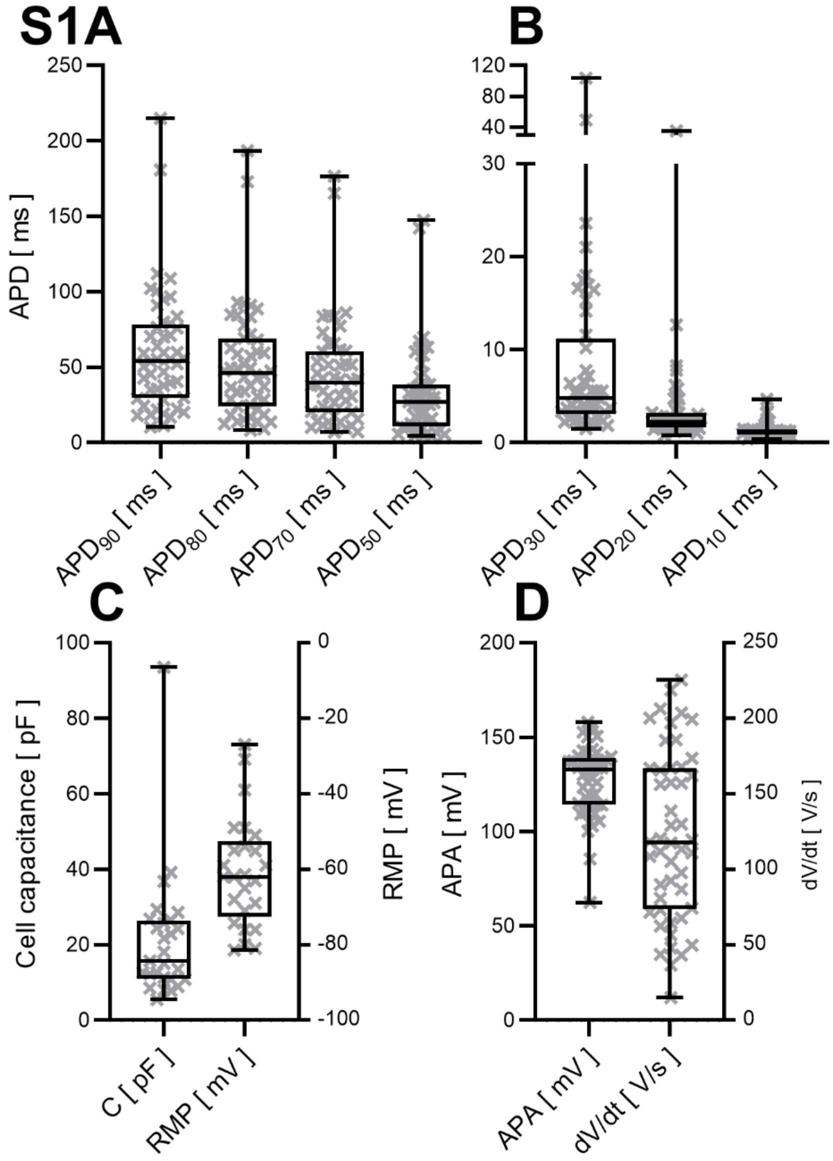

Supplementary figure S1: AP parameters in NRVCM – original data
Chosen AP parameters of the NRVCM analyzed in this work are depicted. Box plots (median, IQR, whiskers from minimum to maximum) and scatter dot plots are shown.

Supplementary table 2: Biological and AP parameters in NRVCM (original data)
Selected biological and AP parameters of the original data in NRVCM are summarized in this table. For better comparability with literature data, both mean ± SEM and median with first and third quartile (Q_1 and Q_3) are provided.

	**Mean ± SEM (or stated parameter)**	**Median, Q_1 – Q_3**
Animal age at isolation [d]	1 – 3 days	-
NRVCM time in culture [d]	1- 4 days (1.7 ± 0.1 days)	-
Spontaneous beating [%]	20.8%	-
Capacity [pF]	21.2 ± 3.3	15.7, 10.9 – 26.5
RMP [mV]	-60.7 ± 2.9	62.0, 52.5 – 72.7
APD_{10} [ms]	1.3 ± 0.1	1.1, 0.8 – 1.4
APD_{20} [ms]	3.6 ± 0.7	2.2, 1.6 – 3.2
APD_{25} [ms]	6.0 ± 1.6	3.2, 2.1 – 5.1
APD_{30} [ms]	9.9 ± 2.3	4.8, 3.1 – 11.2
APD_{40} [ms]	23.1 ± 3.6	16.5, 7.7 – 29.8
APD_{50} [ms]	32.8 ± 29.8	26.8, 10.8 – 38.5
APD_{70} [ms]	45.3 ± 5.1	39.8, 20.3 – 60.6
APD_{75} [ms]	48.3 ± 5.2	42.7, 21.9 – 64.8
APD_{80} [ms]	51.5 ± 5.4	46.2, 24.1 – 68.9
APD_{90} [ms]	59.7 ± 5.8	54.5, 29.4 – 78.4
APA [ms]	127.2 ± 2.7	132.8, 114.3 – 139.1
dV/dt [V/s]	122.3 ± 8.0	117.7, 73.5 – 167.0

3 Normality tests of action potential parameters in NRVCM

APA, APD_{90}, and APD_{50} from our own data set might not be distributed Gaussian (D'Agostino & Pearson test: APA $p = 0.005$, APD_{90} $p < 0.001$ and APD_{50} $p < 0.001$; Anderson-Darling test: APA with $p = 0.064$, APD_{90} $p = 0.002$ and APD_{50} $p < 0.001$; Shapiro-Wilk test: APA with $p = 0.018$, APD_{90} $p < 0.001$ and APD_{50} $p < 0.001$; Kolmogorov-Smirnov test: APA with $p = 0.024$, APD_{90} $p = 0.061$ and APD_{50} $p < 0.001$), while dV/dt appears to be normal distributed (D'Agostino & Pearson test: $p = 0.045$, Anderson-Darling test: $p = 0.219$, Shapiro-Wilk test: $p = 0.196$, Kolmogorov-Smirnov test: $p > 0.1$).

4 Influence of electrode resistance on AP parameters

While we did not test different electrode resistances in our research, we did not qualitatively observe an effect of electrode resistance on the AP in NRVCM in the analyzed literature, as shown in supplementary figure 2.

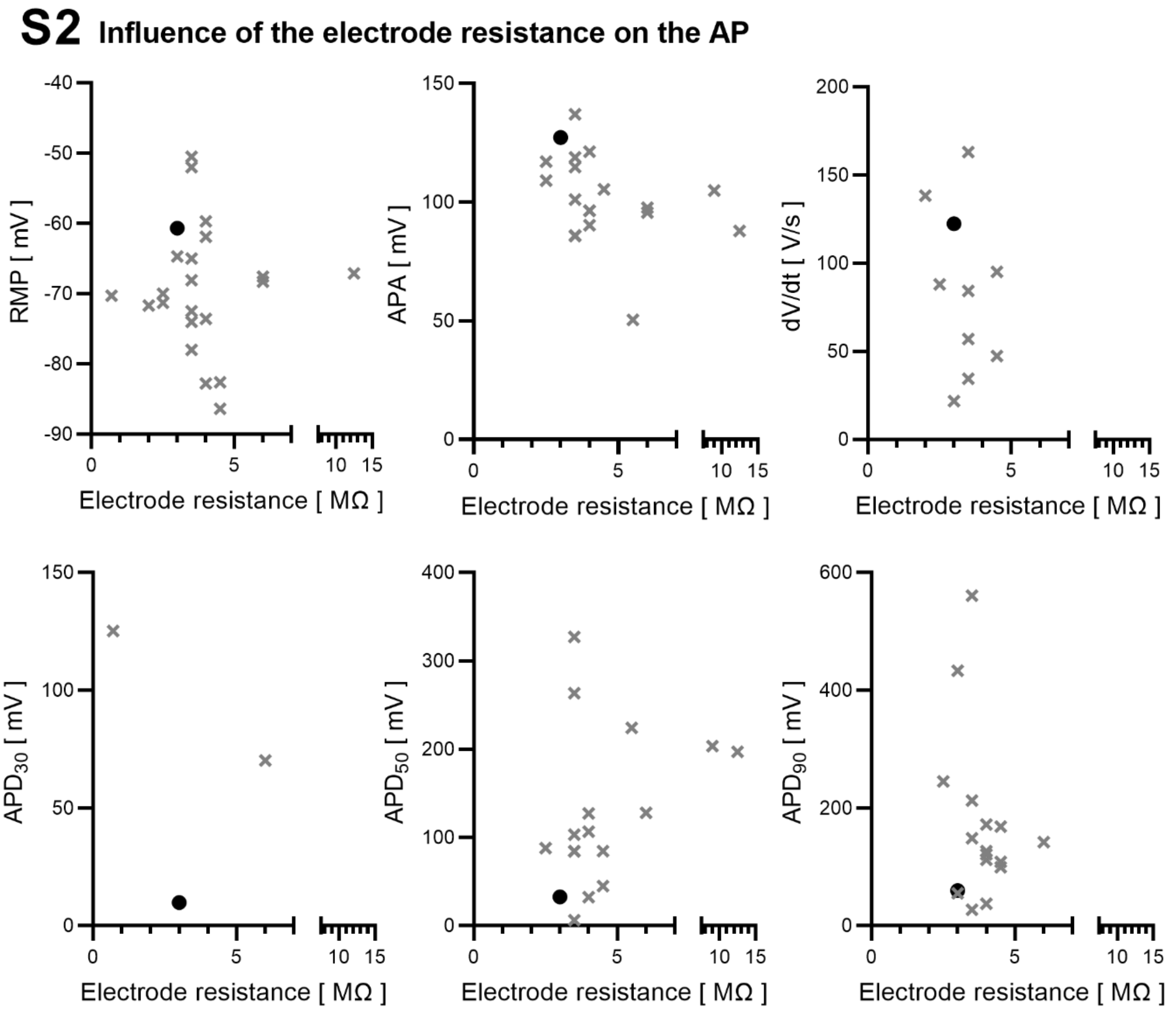

Supplementary figure S2: Influence of the electrode resistance on the AP
Depicted are mean RMP, APA, dV/dt, APD_{30}, APD_{50} and APD_{90} values (grey crosses: data from analyzed literature, black circles: original data) at different electrode resistances.

5 Influence of induction duration on AP parameters

Induction pulse duration does not affect APD_{90}, APD_{50}, APD_{30}, APA or dV/dt and does not qualitatively affect AP shape in NRVCM at current strength close to the individual cells threshold, while it deforms the early phases of the AP at high suprathreshold values due to overlap with the induction pulse, as shown in supplementary figure S2.

Supplementary table 3: Influence of the induction duration on the AP
Mean values ± SEM for APD_{90}, APD_{50}, APD_{30}, APA and dV/dt of individual NRVCMs (n = 5) at different induction current duration (2 ms, 5 ms and 10 ms), are shown. Respective *p*-values (One-way ANOVA with repeated measures as main test, corrected for multiple comparisons with Dunnett's test) for comparison of durations of 5 ms and 10 ms against 2 ms, are shown.

**Duration**	**APD_{90}**	**APD_{50}**	**APD_{30}**	**APA**	**dV/dt**
p value (main test)	0.302	0.198	0.789	0.261	0.721
2 ms	127.8 ± 36.3 ms	61.0 ± 7.8 ms	35.4 ± 7.9 ms	119.1 ± 2.9 ms	62.3 ± 7.4 V/s
5 ms	135.9 ± 36.0 ms	75.2 ± 12.6 ms	38.1 ± 9.8 ms	123.3 ± 4.8 ms	66.6 ± 15.1 V/s
p value	0.104	0.270	0.926	0.561	0.847
10 ms	95.7 ± 17.5 ms	64.4 ± 8.7 ms	41.3 ± 5.4 ms	113.0 ± 6.3 ms	53.6 ± 6.7 V/s
p value	0.403	0.885	0.796	0.425	0.661

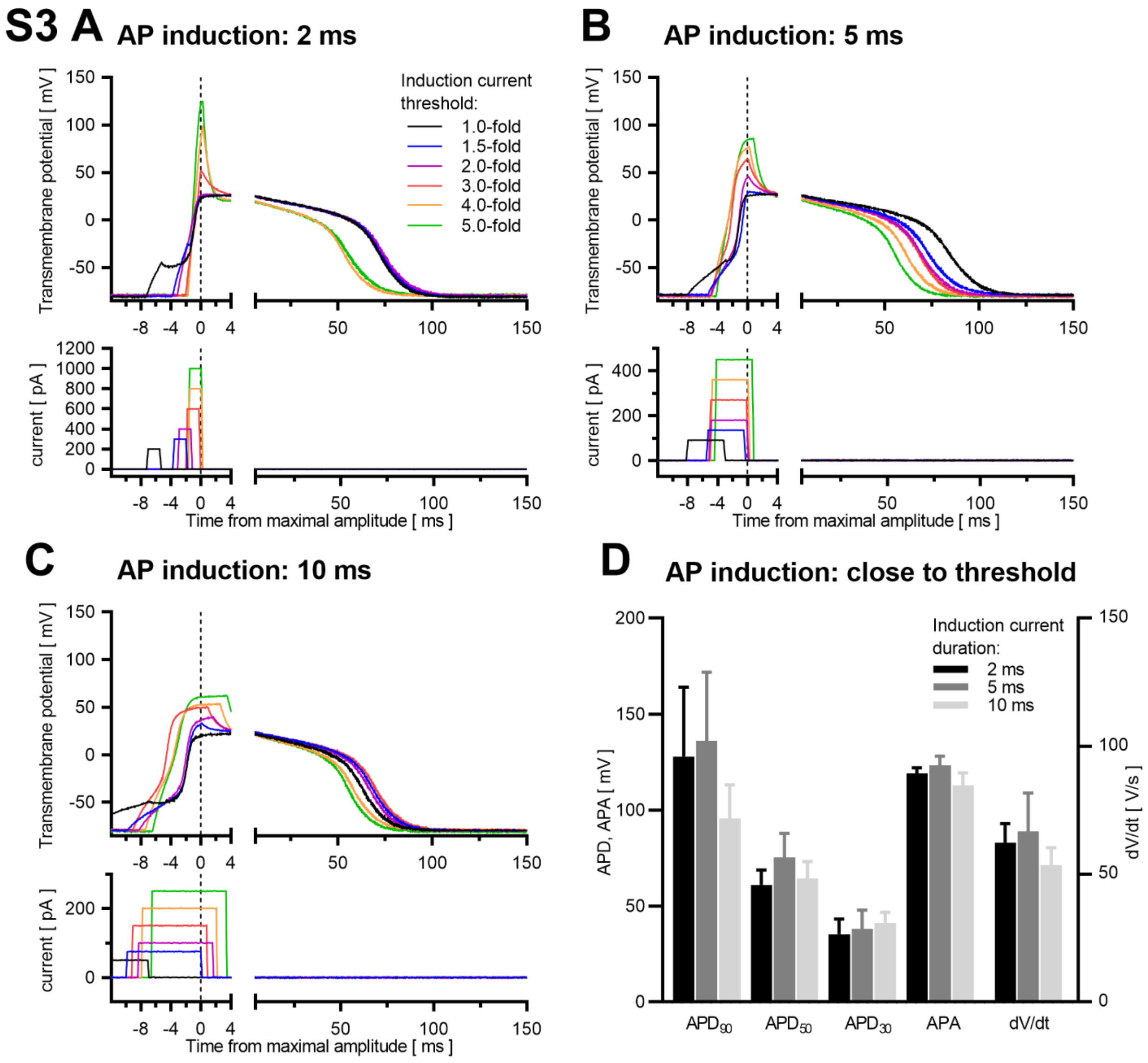

Supplementary figure S3: Influence of the induction duration on the AP

Depicted are exemplary AP waveforms with different induction pulse durations (panel A-C) and different induction pulse amplitudes (1x to 5x suprathreshold). Mean values ± SEM for APD_{90}, APD_{50}, APD_{30}, APA and dV/dt at different induction pulse durations are given in panel D. No significant differences were observed (repeated measures ANOVA).

6 Influence of culture duration of NRVCM on AP parameters

We observed a higher dV/dt with prolongated culture duration and nonsignificant trends towards shorter APDs, higher APA and more negative RMPs with prolongated culture duration. These findings are in accordance with earlier findings (36).

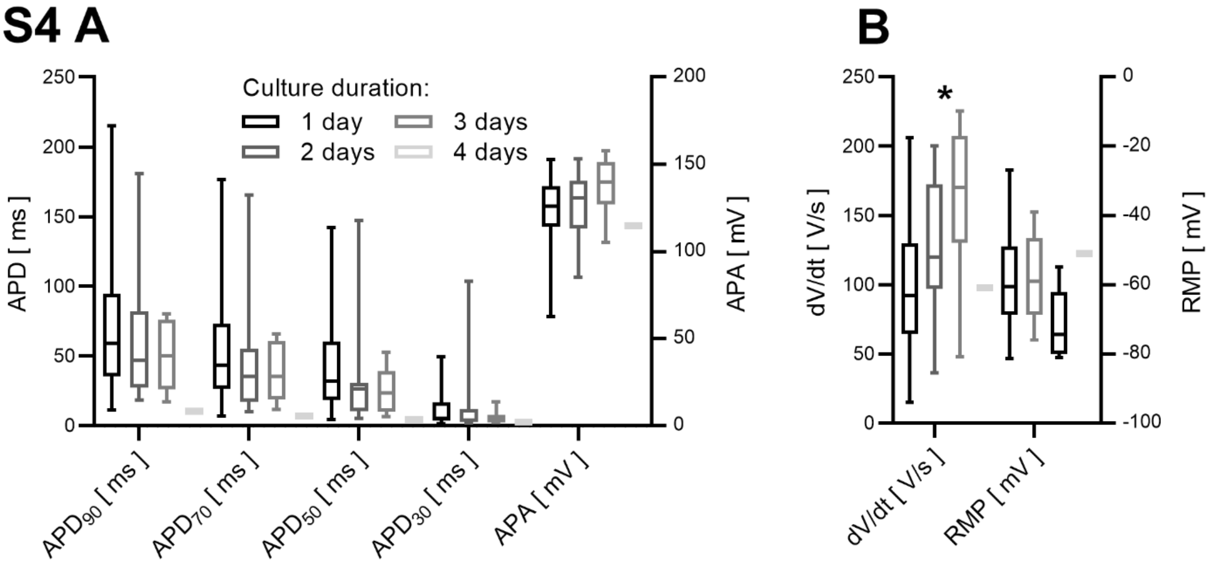

Supplementary figure S4: Influence of culture duration of NRVCM on the AP
Different AP parameters of NRVCM at different culture durations are shown (black: < 24 h (n = 24), dark grey: 24 – 48 h (n = 14), gray: 48 – 72 h (n = 10) and light grey: 72 – 96 h (n = 1)). Box plots (median, IQR, whiskers from minimum to maximum). *: $p < 0.05$ (Kruskal-Wallis test).

Supplementary table 4: Influence of culture duration of NRVCM on the AP
Selected AP parameters of APs in NRVCM are presented regarding the culture duration before AP induction. Median with first and third quartile (Q_1 and Q_3). Statistical analysis: Kruskal-Wallis test.

	**Culture < 24h (n =24) Median, $Q_1 – Q_3$**	**Culture 24-48h (n =10) Median, $Q_1 – Q_3$**	**Culture 48-72h (n =10) Median, $Q_1 – Q_3$**	**Culture 72-96h (n =1)**	***p* value**
APD_{90} [ms]	59.2, 35.5 – 94.7	46.6, 27.4 – 82.0	50.2, 26.2 – 76.1	10.3	0.256
APD_{70} [ms]	43.2, 26.7 – 73.0	35.4, 17.0 – 55.3	35.3, 18.6 – 60.6	6.8	0.265
APD_{50} [ms]	31.9, 18.6 – 60.3	26.4, 10.2 – 30.8	23.5, 9.8 – 39.0	4.6	0.236
APD_{30} [ms]	5.4, 3.7 – 16.6	4.1, 2.6 – 12.2	4.7, 2.3 – 7.9	2.3	0.357
APA [ms]	126.1, 114.1 – 137.4	130.9, 113.0 – 140.6	139.9, 126.9 – 151.4	114.9	0.220
dV/dt [V/s]	92.4, 64.5 – 129.9	119.9, 97.1 – 172.2	170.2, 130.1 – 207.4	97.6	**0.049**
RMP [mV]	-60.5, -68.8 - -49.0	-59.0, -68.7 – 46.5	-74.3, -80 - -62.0	-51.0	0.1869

7 Influence of spontaneously beating *vs* quiescent NRVCM on AP parameters

We observed no significant difference between spontaneously beating *vs* quiescent NRVCM on AP parameters (APD_{90}, APD_{50}, APD_{30}, APA or dV/dt).

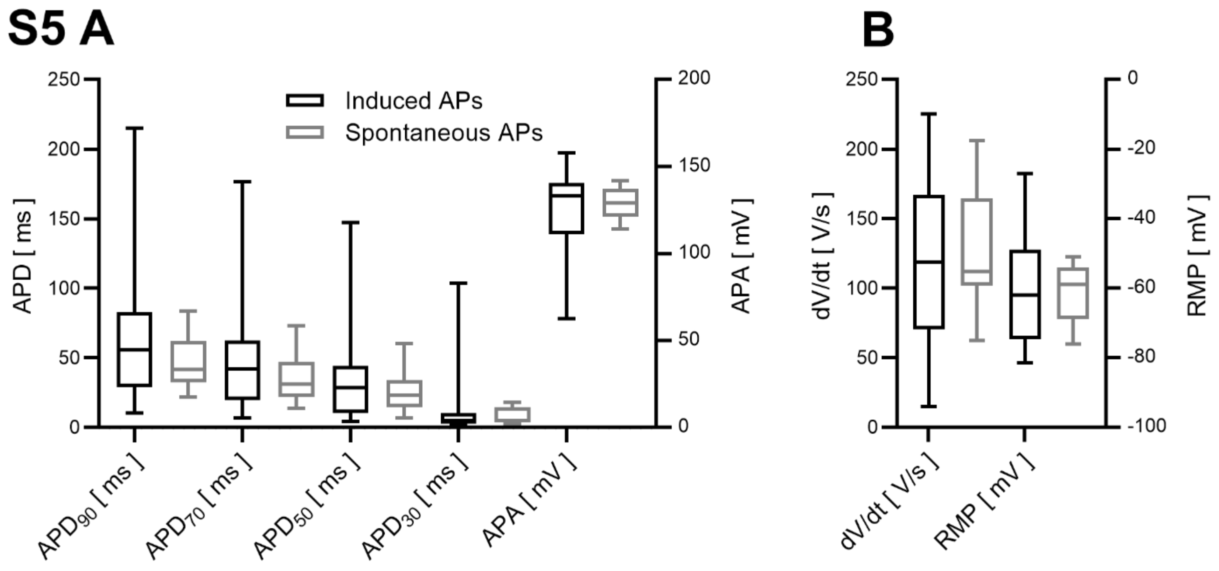

Supplementary figure S5: Influence of spontaneously beating *vs* quiescent NRVCM on the AP Different AP parameters of induced APs in primarily quiescent (black) and spontaneously beating (grey) NRVCM are depicted. Box plots (median, IQR, whiskers from minimum to maximum). No significant differences were observed between both groups (Mann-Whitney *U* test).

Supplementary table 5: Influence of spontaneously beating *vs* quiescent NRVCM on the AP Selected AP parameters of induced APs in primarily quiescent and spontaneously beating (grey) NRVCM are provided. Median with first and third quartile (Q_1 and Q_3). Statistical analysis: Mann-Whitney *U* test.

	**Quiescent (n =38) Median, $Q_1 – Q_3$**	**Spontaneously beating (n =38) Median, $Q_1 – Q_3$**	***p* value**
APD_{90} [ms]	55.7, 29.0 – 82.8	41.5, 32.3 – 62.2	0.334
APD_{70} [ms]	42.1, 19.5 – 62.5	31.0, 22.0 – 47.2	0.401
APD_{50} [ms]	28.6, 10.2 – 44.3	23.2, 14.5 – 34.3	0.591
APD_{30} [ms]	4.8, 2.7 – 10.5	5.0, 3.6 – 14.7	0.626
APA [ms]	133.2, 111.2 – 140.6	129.1, 121.4 – 137.3	0.930
dV/dt [V/s]	118.8, 70.5 – 167.2	112.0, 101.9 – 164.4	0.752
RMP [mV]	-62.0, 49.0 – 74.7	-59.0, 54.0 – 69.0	0.825

8 Median and interquartile range of data

As described in the methods section, we provided several data in the original manuscript as mean + standard error of the mean (SEM), while we assume that our data are not normally distributed. The more appropriate data presentation (median and interquartile range, IQR) for all these data is described in supplementary table 2 and supplementary table 5.

Supplementary table 6: Median and interquartile range of data provided in the manuscript
Data provided in the manuscript as mean + standard error of the mean (SEM) for better comparability with literature data are provided here as median with first and third quartile (Q_1 and Q_3). Compare supplementary table 2 for detailed information on native AP parameters.

**Temperature dependency (section 3.2)**	**28°C (n = 31) Median, $Q_1 – Q_3$**	**37°C (n = 17) Median, $Q_1 – Q_3$**			
APD_{90} [ms]	52.2, 28.3 – 70.4	63.9, 32.4 – 92.8			
APD_{50} [ms]	26.2, 10.1 – 36.5	34.5, 14.3 – 61.6			
APD_{30} [ms]	4.8, 3.0 – 10.2	4.6, 3.1 – 14.7			
APA [ms]	105.1, 64.5 – 165.7	156.1, 112.0 – 167.4			
dV/dt [V/s]	119.7, 109.9 – 137.4	137.9, 129.1 – 151.6			
**HMP dependency (section 3.4)**	**-70 mV (n = 6) Median, $Q_1 – Q_3$**	**-80 mV (n = 7) Median, $Q_1 – Q_3$**	**-90 mV (n = 7) Median, $Q_1 – Q_3$**	**-100 mV (n = 6) Median, $Q_1 – Q_3$**	
APD_{90} [ms]	177.8, 125.7 – 300.8	102.5, 86.8 – 189.2	73.4, 43.8 – 87.0	50.0, 31.5 – 81.5	
APD_{50} [ms]	125.8, 89.1 – 175.8	66.1, 35.0 – 84.4	32.3, 28.5 – 48.0	22.3, 14.6 – 29.3	
APD_{30} [ms]	92.8, 57.2 – 123.6	19.7, 18.4 – 47.2	14.4, 1.2 – 25.6	11.4, 5.3 – 18.5	
APA [ms]	99.0, 93.4 – 112.7	118.4, 105.8 – 137.7	136.0, 127.1 – 145.8	144.9, 133.0 – 152.3	
dV/dt [V/s]	40.5, 29.3 – 88.2	55.8, 47.5 – 99.8	95.5, 69.8 – 131.2	110.7, 73.5 – 161.7	
**Induction dependency (section 3.5)**	**1.0-fold threshold Median, $Q_1 – Q_3$**	**1.5-fold threshold Median, $Q_1 – Q_3$**	**2.0-fold threshold Median, $Q_1 – Q_3$**	**3.0-fold threshold Median, $Q_1 – Q_3$**	**5.0-fold threshold Median, $Q_1 – Q_3$**
APD_{90} [ms]	115.3, 97.4 – 129.7	112.4, 86.9 – 193.8	111.3, 76.8 – 116.5	91.2, 76.6 – 108.2	86.2, 71.8 – 105.5
APD_{50} [ms]	79.7, 66.5 – 100.7	76.6, 51.0 – 79.1	66.1, 55.8 – 73.3	54.3, 26.8 – 57.3	22.7, 10.7 – 36.8
APD_{30} [ms]	49.0, 18.1 – 68.0	38.4, 9.1 – 51.8	18.2, 2.0 – 31.6	4.8, 3.2 – 11.4	1.9, 1.0 – 2.0
APA [ms]	116.6, 108.4 – 128.2	126.7, 114.3 – 133.2	137.7, 126.7 – 139.2	152.1, 144.5 – 162.2	178.8, 167.9 – 190.2
dV/dt [V/s]	68.1, 48.0 – 114.7	68.9, 55.6 – 114.4	78.9, 58.4 – 117.7	74.6, 64.5 – 116.0	88.5, 49.3 – 112.5
